# Enhancing integration of the dairy and beef sectors through application of assisted reproductive technologies: pregnancy outcomes following timed AI and timed ET in lactating dairy cows

**DOI:** 10.1590/1984-3143-AR2025-0055

**Published:** 2025-09-15

**Authors:** Pat Lonergan, Alan Crowe, Laura Thompson, Eliza Murphy, Stephen Butler

**Affiliations:** 1 School of Agriculture and Food Science, University College Dublin, Dublin, Ireland; 2 Teagasc, Animal and Grassland Research and Innovation Centre, Moorepark, Fermoy, Co. Cork, Ireland

**Keywords:** pregnancy loss, IVF, bovine

## Abstract

Assisted reproductive technologies, particularly sex-sorted semen and in vitro embryo production (IVP) can contribute to accelerating genetic gain in both dairy breeds and beef breeds suitable for mating with dairy cows by increasing the number of offspring produced from genetically elite dams. Use of sexed semen has rapidly increased in recent years, accelerating herd genetic gain through selection of the best genetic merit dams to breed replacements, allowing non-replacement dams to be bred to beef sires or to act as recipients of beef embryos to improve calf marketability. IVP offers significant advantages over traditional multiple ovulation embryo transfer (MOET) including increased flexibility in sire usage allowing multiple pregnancies from elite dam-sire combinations to be generated, the ability to produce more embryos per unit time per genetically elite female, the ability to use oocytes from prepubertal females and the more efficient use of rare or high-cost semen straws. Despite these benefits, significant challenges relating to pregnancy loss after embryo transfer, particularly after cryopreservation of IVP embryos, and issues relating to peri- and postnatal health and development of IVP offspring remain to be resolved and hamper the more widespread application of the technology. Improving our understanding of the underlying physiological and molecular mechanisms that regulate early embryo development, embryo-endometrial interactions and lead to successful pregnancy establishment is necessary to understand and elucidate the causes of pregnancy loss and provide a basis for new strategies to improve pregnancy outcomes and reproductive efficiency.

## Introduction

Reproductive efficiency is the cornerstone of all animal-based agricultural enterprises and is crucial for profitable, environmentally sustainable, food systems. With increasing global scrutiny of livestock production systems, the dairy and beef industries face mounting pressure to enhance sustainability, animal welfare, and the efficient use of resources. A central focus is on the production of high-quality animal-derived food products in ways that address both environmental and ethical concerns. Reproductive biotechnologies offer a promising pathway to improving the productivity and environmental performance of seasonal-calving dairy systems, which are designed to optimize pasture-based inputs for both milk and beef production. Innovations in breeding strategies—particularly the use of sex-sorted semen and in vitro embryo production—are being increasingly implemented to enhance genetic gain, improve reproductive efficiency, and mitigate welfare concerns associated with the low economic value and management of surplus male dairy calves (Ritter et al., 2019; Haskell, 2020).

Dairy and beef production systems are inextricably linked, with the integration of “beef-on-dairy” strategies becoming increasingly prominent (Berry, 2021). Surplus calves from the dairy herd have always been an important component of the beef sector. Historically, all dairy females—cows and heifers—were inseminated using semen from dairy breed bulls. A predetermined number of heifer calves were retained as herd replacements, while the remaining surplus calves, predominantly male, were typically sold at low economic value. Advances in reproductive technologies and genetic selection have enabled producers to selectively breed only genetically superior females to generate replacements. This targeted approach facilitates the strategic use of beef semen on lower-genetic-merit animals, thereby improving the terminal value and marketability of non-replacement progeny for beef production. The importance of improving beef production efficiency is pertinent as approximately 60-65% of beef output in the EU now comes from dairy progeny, highlighting the increasing emphasis on the sustainability of dairy calf-to-beef production systems.

Advancements in reproductive technologies including the use of sex-sorted semen and in vitro-produced (IVP) embryos, and better beef sire selection offer opportunities for enhanced dairy-beef production efficiency (Berry, 2021; Crowe et al., 2021). This review summarises data arising from several recent field trials conducted in Ireland examining fertility and pregnancy outcomes in lactating dairy cows following timed artificial insemination (TAI) or timed embryo transfer (TET) with fresh or frozen IVP embryos.

## Welfare concerns and the role of sex-sorted semen

The management of surplus male calves remains a major welfare issue within the dairy industry, largely due to their poor economic value stemming from inferior beef characteristics. This issue is exacerbated in seasonal systems where breeding and calving are concentrated within short windows, resulting in large numbers of male dairy calves being born at the same time, leading to market saturation and ethical concerns regarding their treatment (Crowe et al., 2021; Maher et al., 2021; Webb et al., 2023). The use of sex-sorted semen undoubtedly provides a solution (Holden and Butler, 2018; Butler et al., 2023); by targeting sex-sorted semen toward genetically superior females, dairy producers can efficiently generate high-quality replacement heifers while using beef semen or beef embryos on the remainder of the herd to enhance the growth and carcass quality of non-replacement calves and hence calf marketability.

Paradoxically, the widespread use of sex-sorted semen in dairy herds to generate replacement offspring could hinder the rate of genetic gain as a result of a marked reduction in the births of elite genetic merit male dairy calves that could potentially become future AI sires. In addition, there will be a greater requirement for semen from beef breed bulls that are suitable for crossing with dairy dams to generate the non-replacement calves (Berry and McCarthy, 2025). The use of assisted reproductive technologies (ART), such as multiple ovulation embryo transfer (MOET) and in vitro embryo production, on elite genetic merit dams in both dairy and beef breeds can be harnessed to accelerate genetic gain for both dairy traits (replacement offspring) and beef traits (non-replacement offspring).

## The ART of in vitro embryo production – a tool for genetic improvement

In vitro embryo production is now an established technology in the toolbox of assisted reproductive technologies available to farmers and breeding companies for genetic improvement in dairy cow herds. The number of IVP bovine embryos transferred annually has increased year-on-year in the last decade and now surpasses the number derived by traditional superovulation, accounting for approximately 80% of all bovine embryos produced and transferred globally (Viana, 2023). According to data collated by the International Embryo Technology Society (IETS, 2025), over 1.6 million IVP bovine embryos were transferred commercially worldwide in 2022 compared to ~394,000 in vivo-derived embryos, and this number is predicted to continue to increase (Viana, 2023). IVP offers significant advantages over MOET including increased flexibility in sire usage allowing multiple pregnancies from elite dam-sire combinations to be generated, the ability to produce more embryos per unit time per genetically elite female, the ability to use oocytes from prepubertal females (as young as 2 months of age) to reduce the generation interval (so-called ‘Juvenile In Vitro Fertilization Embryo Transfer’, JIVET) (Baldassarre, 2021; Murphy et al., 2024a) and the more efficient use of rare or high-cost semen straws (e.g., sex-sorted semen) as many oocytes can be inseminated with one semen dose (Crowe et al., 2021). The increased use of sex-sorted semen to breed replacements and beef semen to improve calf quality in dairy herds (Ettema et al., 2017) will ultimately lead to a reduction in the pool of male dairy calves available as future potential AI sires (Butler et al., 2023). Under such conditions, targeted use of IVP with elite breeding stock will facilitate the production of future generations of elite bulls. Despite these major benefits of IVP technology, significant challenges relating to pregnancy loss after embryo transfer, particularly after cryopreservation (freeze/thawing) of IVP embryos, and issues relating to peri- and postnatal health and development of IVP offspring remain to be resolved and hamper the more widespread application of the technology.

## Pregnancy establishment in cattle

Successful establishment of pregnancy in cattle, as in all mammals, requires complex signalling between both the developing conceptus (embryo and extraembryonic membranes) and the uterine endometrium to ensure normal conceptus growth and development (Davenport et al., 2023). Following fertilisation in the oviduct, the early embryo undergoes the first mitotic cleavage divisions before entering the uterus at around the 16-cell stage on approximately Day 4 after ovulation. It soon forms a morula and, by Day 7, a blastocyst, containing an inner cell mass (ICM) and a single layer of trophectoderm surrounding a fluid-filled blastocoel cavity. In contrast to human and rodent embryos which implant soon after hatching from the zona pellucida, in cattle and other ruminants (sheep, goat) the conceptus remains free-floating for the first 2 to 3 weeks before implantation. After hatching on approximately Days 8-9, the spherical blastocyst grows and changes in morphology from a spherical to ovoid shape during a transitory phase preceding the characteristic elongation of the trophectoderm to a filamentous form that begins between Days 12 and 14 (Hue et al., 2012). The ICM differentiates to form the epiblast (which gives rise to the embryo proper) and the hypoblast (which gives rise to the yolk sac) while the trophectoderm gives rise to the placenta. As the conceptus (embryo and associated extraembryonic/placental tissues) elongates and attaches to the endometrial luminal epithelium (LE), on around Day 20 in cattle, it synthesizes and secretes type 1 interferon tau (IFNT), the signal for maternal recognition of pregnancy in ruminants (Bazer and Thatcher 2017; Forde and Lonergan, 2017). IFNT acts on the endometrial LE and superficial glandular epithelium to inhibit luteolytic pulses of prostaglandin F2α that would result in the structural and functional termination of the ovarian corpus luteum. The result is maintenance of the corpus luteum, the source of progesterone which is unequivocally required for the maintenance of pregnancy (Lonergan and Sanchez, 2020). Aberrations during the period of conceptus elongation are strongly implicated in subsequent embryo loss as developmental programs establish the different embryonic and extra-embryonic lineages that continue to differentiate over time (Mamo et al., 2011; Pfeffer and Pearton, 2012; van Leeuwen et al., 2015; Scatolin et al., 2024).

Mononucleate cells (MNC) constitute the majority of the trophoblast cells in the elongating conceptus, are responsible for synthesis and secretion of IFNT and are involved in attachment of the conceptus to the uterine LE for implantation. Binucleate trophoblastic giant cells (BNC) begin to differentiate from the MNC between Days 17 and 19 of gestation and comprise 15-20% of the trophoblast cells during apposition and attachment phases of implantation. Amongst other molecules, these cells secrete pregnancy-associated glycoproteins (PAG). After firm attachment to the LE by Days 20-22, BNC fuse with individual LE cells to form trinucleate fetal-maternal syncytial cells such that these molecules can be released into the uterine vasculature. Indeed, concentrations of PAG in serum can be reliable used to assess BNC development and diagnose pregnancy in cows from approximately Day 25-28 (Sasser et al., 1986) and concentrations are low on Day 28 in cows predicted to undergo late embryonic mortality (Pohler et al., 2016).

## Pregnancy outcomes following transfer of IVP embryos

We recently carried out a large controlled field trial to compare pregnancy success and pregnancy losses in lactating Holstein-Friesian cows that received TAI or TET using fresh or frozen IVP embryos from either dairy or beef breeds (Crowe et al., 2024b). A total of 1,106 cows were enrolled, with 863 receiving ET and 243 undergoing AI. Oocytes were collected from elite dairy and beef donors weekly (without exogenous gonadotropin stimulation), as well as from commercial beef heifers (post-slaughter). Pregnancy rates on Day 32 were similar between AI (48.8%) and ET (48.9%) and between dairy and beef embryos (50.3% vs. 48.1%, respectively), but significantly less for cows receiving frozen embryos (41.6%) compared with fresh embryos (56.1%). However, pregnancy loss between Days 32 and 62 was significantly greater for ET (15.1%) than AI (4.7%). Consistent with the important role of progesterone in endometrial function and conceptus development, serum progesterone (P4) concentrations on Day 7 (i.e., the day of ET or 7 days after AI) were positively associated with pregnancy success; cows in the quartile with the least serum P4 concentrations (quartile 1) had less probability of being pregnant on d 32 (33.4%) compared with cows in the 3 upper quartiles (45.7%, 55.6%, and 61.2% for quartile 2, quartile 3, and quartile 4, respectively). Interestingly, sex ratio (male: female) at d 62 was skewed toward more male fetuses following ET (61.1:38.9) compared with AI (43.2:56.8) and was consistent with the sex ratio among IVP blastocysts (61.2:38.8), indicating that the deviation was due to the sex of the embryos transferred rather than preferential survival of male embryos post-transfer.

A subsequent study (Murphy et al., 2024b) compared pregnancy per embryo transfer (P/ET) following transfer in lactating dairy cows of IVP dairy and beef embryos generated using conventional (i.e., unsorted) or sex-sorted semen for in vitro fertilization (IVF). Blastocyst yield and the number of blastocysts produced per IVF were similar for conventional (20.0% and 1.7) and sex-sorted (24.7% and 2.1) semen. Pregnancy per ET on d 32 and d 63 were not different between dairy and beef embryos (d 32: 56.6% and 59.4%; d 63: 49.5% and 53.4%, respectively) or between embryos derived from conventional and sex-sorted semen (d 32: 57.5% and 58.6%; d 63: 48.8% and 54.1%, respectively). Pregnancy loss between d 32 and d 63 was not different between dairy and beef embryos or between embryos derived from conventional sex-sorted semen.

## Pregnancy loss from IVP embryos

In cattle, most pregnancy failure occurs quite early after fertilisation; ~75% of conceptus loss occurs in the first 2-3 weeks of gestation, before maternal recognition of pregnancy (around Day 16-17) and the start of placentation (around Day 20) (Sartori et al., 2010; Forde et al., 2011; Wiltbank et al., 2016; Berg et al., 2022; Crowe et al., 2024a). Indeed, in some situations (e.g., metabolic stress associated with high milk production), as many as 50% of embryos may be lost in the first week after fertilisation (Sartori et al., 2010; Berg et al., 2022). However, even when all of the biological and technical causes for pregnancy failure in the first week are avoided by transferring an embryo directly into the uterus (typically done on Day 7 of the cycle), pregnancy success is not consistently improved compared to artificial insemination (Hansen, 2020). This greater embryo mortality presents an obstacle to more widespread use of IVP embryos (Ealy et al., 2019). Thus, improving our understanding of the underlying physiological and molecular regulation of early embryo development leading to a successful pregnancy will significantly contribute to social and economic sustainability in agri-food production, a crucial objective in the face of an ever-increasing global population (United Nations, 2016) and growing concerns about the impact of inefficient agricultural practices on the environment (European Commission, 2019).

Approximately 80-90% of immature bovine oocytes submitted to IVP undergo nuclear maturation in vitro and reach metaphase II, about 80% undergo fertilization, 30-50% develop to blastocyst stage, and around 50% of transferred embryos establish a pregnancy (Lonergan and Fair, 2008; Hansen, 2020). Improvements in culture media and IVP processes have improved initial pregnancy rates to the extent that they are now comparable with AI when transferred fresh; however, subsequent pregnancy loss, particularly from frozen-thawed IVP embryos, remains an obstacle to their more widespread use (Ealy and Seekford, 2019; Hansen, 2020; Crowe et al., 2024a, b). The underlying mechanisms responsible for such loss are not clear but are likely related to the consequences of suboptimal post-fertilisation culture conditions on blastocyst quality (Rizos et al., 2002; Lonergan et al., 2003a, b). IVP embryos differ in terms of morphology, ultrastructure, cryotolerance, and transcriptome from those derived in vivo (Lonergan and Fair, 2008) leading to a compromised ability of the resulting conceptus to appropriately signal to the maternal endometrium during elongation and attachment (Bauersachs et al., 2009; Mansouri-Attia et al., 2009; Sanchez et al., 2019). Appropriate molecular interaction between conceptus and endometrium is an important feature in the period leading up to attachment (Moraes et al., 2018; Sanchez et al., 2019; Biase et al., 2023), and dysregulation of conceptus-maternal communication is a probable cause of lower calving rates after the transfer of IVP embryos. For example, delayed conceptus elongation results in short conceptuses which secrete much less interferon tau (Rizos et al., 2012) and fail to elicit an appropriate transcriptional response from the endometrium compared to long conceptuses (Sanchez et al., 2019).

**Figure 1:**
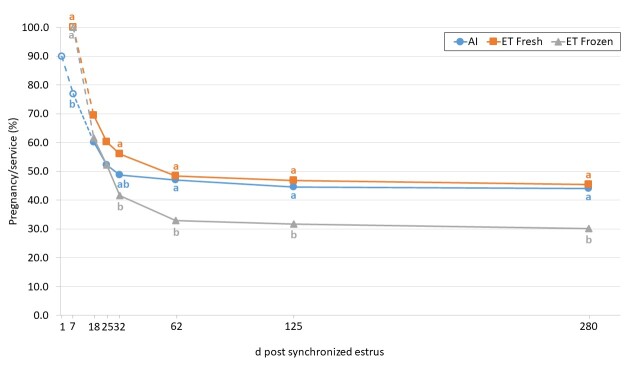
Predicted probability of pregnancy/service following timed AI or timed embryo transfer (ET) with fresh or frozen in vitro-produced embryos. Values not sharing a common letter (a-b) differ (P < 0.05). Open shapes and dashed lines indicate assumed pregnancy results.

**Figure 2:**
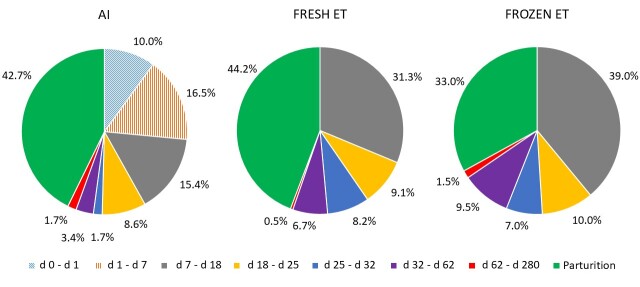
Incidence and timing of pregnancy loss following timed AI or timed embryo transfer (ET) with fresh or frozen in vitro-produced embryos. Percentage pregnancy loss within the windows of Day 0 to 1 (AI only), Day 1 to 7 (AI only), Day 7 to 18, Day 18 to 25, Day 25 to 32, Day 32 to 62, Day 62 to 280, and the percentage of cows that reached parturition at full-term are indicated. Pregnancy was diagnosed by measurement of mRNA abundance of interferon-stimulated gene 15 in peripheral blood on Day 18, serum pregnancy-specific protein B concentration on Day 25 and ultrasound scanning on Day 32, 62, and 125. For cows that received AI, the hatched quadrants are based on assumed pregnancy on Day 0, 1, and 7.

Consistent with data reviewed by Sartori et al. (2010) and Wiltbank et al. (2016), Berg et al. (2022) reported that the first week following insemination is the period of major pregnancy loss in pasture-based dairy cows. They reported pregnancy rates of 71% (d 7), 59% (d 15), 64% (d 28), 62% (d 35), and 57% (d 70); fertilization failure accounted for 16% of failures while development arrest before the morula stage and elongation failure also contributed. Our recent data (Crowe et al., 2025b) (Figures 1 and 2) characterized the incidence and timing of pregnancy loss from service event (TAI or TET with a fresh or frozen IVP blastocyst) to parturition in lactating Holstein-Friesian cows. Consistent with the previous publications above, the data illustrate that most embryonic loss occurs early after fertilization, irrespective of whether AI or ET is used. The predicted probability of pregnancy (%) varied between treatments at each time point (d7, 18, 25, 32, 62, 125, parturition) depending on treatment (AI: 77.0, 60.2, 52.3, 48.8, 47.0, 44.6, 44.0; fresh ET: 100.0, 69.5, 60.3, 56.1, 48.4, 46.8, 45.5; frozen ET: 100.0, 61.7, 52.2, 41.6, 32.9, 31.8, 30.2) (Figure 2). Irrespective of treatment, the largest proportion of pregnancy loss occurred in the period from service event (AI on d 0 or ET on d 7) to d 18. There was greater probability of pregnancy loss between d 32 and 62 following ET (fresh: 11.3%, Frozen: 18.0%) than AI (4.0%), with minimal loss occurring between d 62 and parturition (AI: 1.8%, fresh ET: 1.9%, frozen ET: 3.5%). Treatment differences in the predicted probability of pregnancy were detected between fresh ET versus frozen ET on d 32 and both AI and fresh ET versus frozen ET on d 62, 125, and at parturition. The percentage of cows that calved following transfer of a fresh embryo (45.5%) was similar to AI (44.0%), but less when a frozen embryo was transferred (30.2%).

**Source:**Crowe et al. (2025b).

**Source:**Crowe et al. (2025b).

## Timing of conceptus attachment as a cause of pregnancy loss

Conceptus attachment in cattle is estimated to begin between d 18 and 22 after AI (Wathes and Wooding, 1980; Guillomot and Guay, 1982). During this period, trophoblast giant cells migrate and fuse with the uterine surface epithelium. These cells produce pregnancy-associated glycoproteins, including pregnancy-specific protein B (PSPB), which migrates from the conceptus across the newly forming placenta into maternal circulation (Wooding, 2022). Recent studies indicated that measurement of circulating concentrations of PSPB in individual cows during the expected time of conceptus attachment could accurately detect nonpregnant cows; failure to detect a 10% or greater increase in serum PSPB relative to a preattachment baseline concentration on d 17 accurately diagnosed nonpregnancy at 24 d post-AI (Middleton and Pursley, 2019). Stangaferro et al. (2021) used a combination of plasma PSPB measurements on d 22, 25, 29, and 32 and transrectal ultrasound to estimate pregnancy and pregnancy loss following TAI. Compared with cows that lost their pregnancy by d 32 after TAI, cows that remained pregnant had greater circulating concentrations of PSPB on d 25, 29, and 32 after TAI. Middleton et al. (2022) and Santos et al. (2023) reported that daily serum PSPB measurements could pinpoint the specific day when presumptive conceptus attachment (pCA) occurred. Interestingly, both studies reported associations between greater parity and delayed pCA, and Middleton et al. (2022) reported that nulliparous heifers had earlier pCA compared with both primiparous and multiparous cows. Importantly, these data support the time to pCA as a direct determinant of subsequent pregnancy loss in lactating dairy cows; cows that had a delayed increase in PSPB (on d 22 or after) exhibited a marked increase in the likelihood of embryonic mortality (Santos et al., 2023).

In collaboration with the group at Michigan State University, we carried out a study aimed at determining the timing of pCA and subsequent incidence of pregnancy loss in seasonal-calving pasture-based lactating dairy cows following TAI with conventional (TAI-C) or X-sorted (TAI-S) semen or TET with a frozen-thawed IVP embryo (Crowe et al., 2025c). Lactating cows were synchronized with a 10-d Progesterone-Ovsynch protocol, and were either inseminated 16 h after the second GnRH or received TET on d 7 after presumptive estrus. For all cows that had not returned to estrus, serum PSPB was measured on d 7, 17, and daily from d 19 through 28 after expected ovulation to characterize the timing of pCA. The day of pCA was defined as the first day of an increase in PSPB of ≥12.5% from baseline (d 17) followed by 2 more consecutive days of ≥12.5% increase from the previous day. Pregnancy was diagnosed in cows that had not returned to estrus via ultrasound examination on d 32, 62, and 120 post-ovulation, and calving data were recorded. Day of pCA (mean; 95% CI) was earlier for TAI-C (20.0; 19.7, 20.3) compared with TET (20.6; 20.3, 20.9), and TAI-S (20.3; 19.9, 20.6) was not different from the other 2 treatments. Calving/service event was greater (83.2% vs. 54.4%) and pregnancy loss during the interval from pCA to expected calving date was less (16.8% vs. 45.6%) for cows with early pCA (≤d 20; 23/137) compared with cows that had late pCA (≥d 21; 36/79). The incidence of pregnancy loss was greater for cows assigned to TAI-S (30.7%) and TET (33.8%) than TAI-C (16.4%). These data suggest that the timing of pCA is at least one factor contributing to greater loss of IVP pregnancies.

## JIVET

The ovaries of young animals are characterized by much greater numbers of antral follicles compared with older donors (Desjardins and Hafs, 1969), and hence more oocytes are typically recovered from young animals per OPU session (Landry et al., 2016). Although viable embryos can be produced, the success rate of OPU-IVP with prepubertal heifer donors is generally poorer compared with that achieved with postpubertal and mature female donors (Baruselli et al., 2016).

Laparoscopic ovum pickup (LOPU) in prepubertal calves followed by in vitro embryo production and transfer into adult recipients, also known as juvenile IVF embryo transfer (JIVET), has great potential for accelerated genetic gain through significant shortening of the generation interval. This allows the production of progeny from prepubertal females as young as 2 to 6 mo of age (Baldassarre, 2021). JIVET exploits the fact that, although prepubertal females are incapable of ovulation, waves of follicular growth occur and the recruited follicles can be stimulated with exogenous gonadotropins to produce competent oocytes for aspiration, followed by in vitro embryo production (Currin et al., 2017; Baldassarre and Bordignon, 2018). In contrast to adult donors, hormonal stimulation of prepubertal donors is critical given the fact that their hypothalamus-pituitary-ovary axis is not yet fully functional. Oocytes collected from 2- to 6-mo-old Holstein calves exhibited greater rates of development to the blastocyst stage following longer gonadotropin stimulation (3 d) compared with either shorter duration (2 d) or no stimulation, which was associated with a greater proportion of larger follicles (Currin et al., 2017).

We recently investigated the use of JIVET in a seasonal pasture-based dairy production system (Murphy et al., 2025b forthcoming). Elite Holstein Friesian donor calves (n = 49) were enrolled, all of which were within the top 2% of the Economic Breeding Index. On the day of LOPU, mean (±SD) calf age was 79.6 ± 9.9 days (range: 55-102). Immature cumulus-oocyte complexes (COCs) were collected from each donor on one occasion following FSH stimulation. IVF was conducted using X-sorted semen from a panel of eight Holstein Friesian bulls and following 7 days of in vitro culture, a single blastocyst (grade 1, n = 40; grade 2, n = 34; grade 3, n = 13) was transferred fresh to synchronised recipients (n=87; heifers, n=77; lactating dairy cows, n=10). Mean (±SD) number of COCs recovered per donor was 14.9 ± 15.4 (range, 1-71), and the mean number of transferable embryos produced per donor was 1.77 ± 2.9 (range, 0-15). Mean cleavage and blastocyst rates following IVF were 65.0 ± 26.7% and 12.8 ± 18.6%, respectively. Across all embryos transferred, pregnancy/ET on Day 35 was 39.1% (34/87) and 33.3% (29/87) on Day 62-64. The pregnancy/ET for grade 1, grade 2, and grade 3 embryos on Day 35 was 47.5% (19/40), 38.2% (13/34), and 15.4% (2/13), respectively, and the corresponding values on Days 62-64 were 45% (18/40), 29.4% (10/34), and 7.7% (1/13), respectively. Pregnancy loss between Day 35 and Day 62-64 was 14.7%.

## Calf characteristics following IVP

Using data from the study of Crowe et al. (2024b), we examined the effect of embryo origin (AI vs. IVP), calf breed (beef vs. dairy), and calf sex on gestation length (GL), birthweight (BW), calving difficulty (CD) score and perinatal mortality (Crowe et al., 2025a forthcoming). Data were obtained from 442 calves. For subsets of these calves, birth weight was recorded immediately after birth (n = 281), blood samples were collected at birth for biochemical and hematological analysis (n = 108), and at 24 h after birth to measure serum immunoglobulin G concentration in order to assess passive transfer of immunity (n =126). Overall, GL (mean days, 95% CI) was shorter for calves derived from AI (278.9; 277.0, 280.8) compared with calves derived from fresh ET (281.8; 279.8, 283.7) and frozen ET (282.0; 280.0, 284). Within the population of Holstein Friesian calves (n = 159), BW (mean kg, 95% CI) was lighter for calves derived from AI (34.1 kg; 32.3, 35.9) compared with fresh ET (40.5 kg; 39.0, 42.1) and frozen ET (39.3 kg; 37.7, 41.1). Amongst calves derived from ET, breed affected GL, BW and CD: calves that were sired by the Limousin bull had a longer GL (290.9 d) than Angus (282.0 d), Holstein Friesian (280.5 d) and Jersey calves (282.0 d). Angus calves were heavier (Fresh: 46.3 kg, Frozen: 43.5 kg) than both Holstein Friesian (Fresh: 39.7 kg, Frozen: 38.5 kg) and Jersey calves (Fresh: 30.8 kg, Frozen: 28.8 kg). Angus calves derived from fresh ET had greater mean CD score than Holstein Friesian calves derived from both AI and fresh ET. Results of biochemical and hematological analyses were within the normal range for healthy calves. Calf origin did not affect passive transfer of serum immunoglobulin G from maternal colostrum to calf circulation. In summary, calves originating from IVP-ET were heavier at birth, had longer GL and a greater incidence of CD than calves derived from AI. In general, blood measurements of neonatal calf health were not affected by the origin (AI or ET) of the calf and all calves had similar (low) incidence of perinatal mortality.

Using data generated over four years (2021-2025) from the studies described above, Murphy et al. (2025b forthcoming) characterized the main causes of death in calves derived from IVP that had been submitted for postmortem examination (PME). A total of 1592 embryos were transferred resulting in 746 pregnancies that reached full-term; a total of 50 calves were stillborn or died postpartum, of which 44 calves were submitted for PME (Dairy Fresh = 13, Dairy Frozen = 6, Beef Fresh = 19, Beef Frozen = 6 and Dairy JIVET Fresh = 2). Cause of death was categorized as dystocia (n =15), congenital defect (n = 11), infection (n = 5), multiple causes (n = 4), diagnosis not reached (n = 4) and other (n = 5); the latter category included bloat, umbilical haemorrhage, placental abruption and bowel torsion. Time of death was categorized as (i) stillborn or died ≤ 30 minutes after birth (61.4%, 27/44), (ii) >30 mins and ≤ 24 hours after birth (13.6%, 6/44); (iii) > 24 hours to ≤ 7 days after birth (9.1%, 4/44); (iv) 8 to 21 days after birth (6.8%, 3/44), and (v) > 21 days after birth (9.1%, 4/44). Congenital defects identified during PME were classified according to the primary organ affected including intestines (n = 5), heart (n = 1), liver (n = 2), and other (n = 3). Of the 44 calves included in the dataset, 36.4% had birthweight > 50 kg. Of the 11 diagnoses of congenital defect, the organ most commonly affected was the intestines (5), followed by the liver (2) and heart (1). In summary, dystocia and congenital defect were the two primary cause of death identified in calves derived from IVP embryos.

Finally, Thompson et al. (2025) compared the liver and muscle transcriptome in 4-month-old male and female dairy calves conceived by artificial insemination (AI) or by the transfer of an IVP embryo (n = 4 per sex per treatment). Analysis of the RNAseq data revealed a distinct separation between the liver transcriptomes of female and male calves, regardless of method of production. Moreover, within the cohort of female calves, a strong separation between those derived from IVP vs AI was observed. Analysis of differentially expressed genes indicated downregulation of oxidative phosphorylation pathways and upregulation of immune system-related enriched terms, including Th17 cell differentiation and antigen processing and presentation. For the muscle transcriptome, the separation between male and female calves was less apparent compared with the liver transcriptome, but there was still a clear separation between female calves derived from IVP vs AI, with downregulated genes enriching for p53 signalling and upregulated genes enriching terms related to muscle structure development. These findings demonstrate that the IVP process induces significant alterations in the liver and muscle transcriptome of female postnatal calves. The data are consistent with those of Rabaglino et al. (2021) who reported evidence for an early activation of the hypothalamic-pituitary-gonadal axis and altered hepatic and muscular energy regulation in the same male dairy calves (Rabaglino et al., 2022) in phenotypically normal 3-month-old male IVP dairy calves compared with MOET-derived counterparts. The long term consequences of these differences are unclear.

## Conclusion

Reproductive technologies, such as sex-sorted semen and IVP, are transforming how we manage both dairy and beef production. These technologies enable farmers to enhance genetic progress, improve the economic value of their calf crop, and address welfare concerns associated with male dairy calves. While challenges remain, particularly in the implementation of ARTs in seasonal-calving systems, ongoing research and technological advancements are likely to further improve the efficiency and sustainability of both dairy and beef sectors. In an era of increasing scrutiny on the environmental and ethical impacts of livestock farming, the use of these technologies represents a crucial step towards more sustainable and responsible animal production systems. Further work is required to improve the likelihood of pregnancy establishment and reduce embryonic and fetal mortality following transfer of IVP embryos.

## Data Availability

Research data are available in the body of the article and in the original cited articles.

## References

[B001] Baldassarre H, Bordignon V (2018). Laparoscopic ovum pick-up for in vitro embryo production from dairy bovine and buffalo calves. Anim Reprod.

[B002] Baldassarre H (2021). Laparoscopic ovum pick-up followed by in vitro embryo production and transfer in assisted breeding programs for ruminants. Animals.

[B003] Baruselli PS, Batista EOS, Vieira LM, Ferreira RM, Guerreiro BG, Bayeux BM, Sales JNS, Souza AH, Gimenes LU (2018). Factors that interfere with oocyte quality for in vitro production of cattle embryos: effects of different developmental & reproductive stages. Anim Reprod.

[B004] Bauersachs S, Ulbrich SE, Zakhartchenko V, Minten M, Reichenbach M, Reichenbach HD, Blum H, Spencer TE, Wolf E (2009). The endometrium responds differently to cloned versus fertilized embryos. Proc Natl Acad Sci USA.

[B005] Bazer FW, Thatcher WW (2017). Chronicling the discovery of interferon tau. Reproduction.

[B006] Berg DK, Ledgard A, Donnison M, McDonald R, Henderson HV, Meier S, Juengel JL, Burke CR (2022). The first week following insemination is the period of major pregnancy failure in pasture-grazed dairy cows. J Dairy Sci.

[B007] Berry DP, McCarthy J (2025). Sire mating advice framework for cattle to recommend which beef bull to mate to individual dairy females. J Dairy Sci.

[B008] Berry DP (2021). Invited review: Beef-on-dairy-The generation of crossbred beef x dairy cattle. J Dairy Sci.

[B009] Biase FH, Moorey SE, Schnuelle JG, Rodning S, Ortega MS, Spencer TE (2023). Extensive rewiring of the gene regulatory interactions between in vitro-produced conceptuses and endometrium during attachment. Pnas Nexus.

[B010] Butler ST, Crowe AD, Moore SG, Lonergan P (2023). Review: use of assisted reproduction in seasonal-calving dairy herds. Animal.

[B011] Crowe AD, Lonergan P, Butler ST (2021). Invited review: use of assisted reproduction techniques to accelerate genetic gain and increase value of beef production in dairy herds. J Dairy Sci.

[B012] Crowe AD, Sanchez JM, Moore SG, McDonald M, McCabe MS, Randi F, Lonergan P, Butler ST (2024). Incidence and timing of pregnancy loss following timed artificial insemination or timed embryo transfer with a fresh or frozen in vitro-produced embryo. J Dairy Sci.

[B013] Crowe AD, Sanchez JM, Moore SG, McDonald M, Rodrigues R, Morales MF, Freitas LOD, Randi F, Furlong J, Browne JA, Rabaglino MB, Lonergan P, Butler ST (2024). Fertility in seasonal-calving pasture-based lactating dairy cows following timed artificial insemination or timed embryo transfer with fresh or frozen in vitro-produced embryos. J Dairy Sci.

[B014] Crowe AD, Doyle RC, Lonergan P, Butler ST (2025). Gestation length, calf birth weight, calving difficulty, perinatal mortality and calf health following timed artificial insemination or embryo transfer with fresh or frozen in vitro produced embryos. J Dairy Sci.

[B015] Crowe AD, Sanchez JM, Moore SG, McDonald M, McCabe MS, Randi F, Lonergan P, Butler ST (2025). Incidence and timing of pregnancy loss following timed artificial insemination or timed embryo transfer with a fresh or frozen in vitro-produced embryo. J Dairy Sci.

[B016] Crowe AD, Sanchez JM, Moore SG, McDonald M, Randi F, Santos A, Minela T, Branen J, Furlong J, Pursley JR, Lonergan P, Butler ST (2025). Time to presumptive conceptus attachment and subsequent pregnancy loss in pasture-based lactating dairy cows following artificial insemination with conventional or X-sorted semen or embryo transfer. J Dairy Sci.

[B017] Currin L, Michalovic L, Bellefleur AM, Gutierrez K, Glanzner W, Schuermann Y, Bohrer RC, Dicks N, da Rosa PR, De Cesaro MP, Lopez R, Grand FX, Vigneault C, Blondin P, Gourdon J, Baldassarre H, Bordignon V (2017). The effect of age and length of gonadotropin stimulation on the in vitro embryo development of Holstein calf oocytes. Theriogenology.

[B018] Davenport KM, Ortega MS, Johnson GA, Seo H, Spencer TE (2023). Review: implantation and placentation in ruminants. Animal.

[B019] Desjardins C, Hafs HD (1969). Maturation of bovine female genitalia from birth through puberty. J Anim Sci.

[B020] Ealy AD, Seekford ZK (2019). Symposium review: predicting pregnancy loss in dairy cattle. J Dairy Sci.

[B021] Ealy AD, Wooldridge LK, McCoski SR (2019). BOARD INVITED REVIEW: post-transfer consequences of in vitro-produced embryos in cattle. J Anim Sci.

[B022] Ettema JF, Thomasen JR, Hjorto L, Kargo M, Ostergaard S, Sorensen AC (2017). Economic opportunities for using sexed semen and semen of beef bulls in dairy herds. J Dairy Sci.

[B023] European Commission (2019). The European Green Deal.

[B024] Forde N, Carter F, Spencer TE, Bazer FW, Sandra O, Mansouri-Attia N, Okumu LA, McGettigan PA, Mehta JP, McBride R, O’Gaora P, Roche JF, Lonergan P (2011). Conceptus-induced changes in the endometrial transcriptome: how soon does the cow know she is pregnant?. Biol Reprod.

[B025] Forde N, Lonergan P (2017). Interferon-tau and fertility in ruminants. Reproduction.

[B026] Guillomot M, Guay P (1982). Ultrastructural features of the cell surfaces of uterine and trophoblastic epithelia during embryo attachment in the cow. Anat Rec.

[B027] Hansen PJ (2020). The incompletely fulfilled promise of embryo transfer in cattle-why aren’t pregnancy rates greater and what can we do about it?. J Anim Sci.

[B028] Haskell MJ (2020). What to do with surplus dairy calves? Welfare, economic, and ethical considerations. Landbauforschung.

[B029] Holden SA, Butler ST (2018). Review: applications and benefits of sexed semen in dairy and beef herds. Animal.

[B030] Hue I, Degrelle SA, Turenne N (2012). Conceptus elongation in cattle: genes, models and questions. Anim Reprod Sci.

[B031] IETS (2025). IETS.

[B032] Landry DA, Bellefleur AM, Labrecque R, Grand FX, Vigneault C, Blondin P, Sirard MA (2016). Effect of cow age on the in vitro developmental competence of oocytes obtained after FSH stimulation and coasting treatments. Theriogenology.

[B033] Lonergan P, Fair T (2008). In vitro-produced bovine embryos: dealing with the warts. Theriogenology.

[B034] Lonergan P, Rizos D, Gutierrez-Adan A, Moreira PM, Pintado B, de la Fuente J, Boland MP (2003). Temporal divergence in the pattern of messenger RNA expression in bovine embryos cultured from the zygote to blastocyst stage in vitro or in vivo. Biol Reprod.

[B035] Lonergan P, Rizos D, Kanka J, Nemcova L, Mbaye AM, Kingston M, Wade M, Duffy P, Boland MP (2003). Temporal sensitivity of bovine embryos to culture environment after fertilization and the implications for blastocyst quality. Reproduction.

[B036] Lonergan P, Sanchez JM (2020). Symposium review: progesterone effects on early embryo development in cattle. J Dairy Sci.

[B037] Maher JW, Clarke A, Byrne AW, Doyle R, Blake M, Barrett D (2021). Exploring the Opinions of Irish Dairy Farmers Regarding Male Dairy Calves. Front Vet Sci.

[B038] Mamo S, Mehta JP, McGettigan P, Fair T, Spencer TE, Bazer FW, Lonergan P (2011). RNA sequencing reveals novel gene clusters in bovine conceptuses associated with maternal recognition of pregnancy and implantation. Biol Reprod.

[B039] Mansouri-Attia N, Sandra O, Aubert J, Degrelle S, Everts RE, Giraud-Delville C, Heyman Y, Galio L, Hue I, Yang XZ, Tian XC, Lewin HA, Renard JP (2009). Endometrium as an early sensor of in vitro embryo manipulation technologies. Proc Natl Acad Sci USA.

[B040] Middleton EL, Minela T, Ahearne M, Arnold H, Santos A, Pursley JR (2022). Dairy heifers have an earlier increase in serum pregnancy-specific protein B compared with lactating dairy cows. Is this an indicator of earlier conceptus attachment?. JDS Commun.

[B041] Middleton EL, Pursley JR (2019). Short communication: blood samples before and after embryonic attachment accurately determine non-pregnant lactating dairy cows at 24 d post-artificial insemination using a commercially available assay for pregnancy-specific protein B. J Dairy Sci.

[B042] Moraes JGN, Behura SK, Geary TW, Hansen PJ, Neibergs HL, Spencer TE (2018). Uterine influences on conceptus development in fertility-classified animals. Proc Natl Acad Sci USA.

[B043] Murphy EM, Thompson L, McDonald M, Doyle RC, Silva T, Bell L, Chaubal S, Creek M, Herlihy MM, Parra E, Torruella X, Huuskonen V, Randi F, Lonergan P, Butler ST (2025). Juvenile in vitro fertilization embryo transfer in seasonal pasture-based dairy systems..

[B044] Murphy EM, Crowe AD, Thompson L, Moore SG, McDonald M, Hordern E, Bertholdi B, Randi F, Canadas ER, Lonergan P, Butler ST (2025). Fertility in lactating dairy cows following timed embryo transfer with fresh in vitro-produced embryos derived from conventional or sex-sorted semen. J Dairy Sci.

[B045] Murphy EM, Mee JF, Crowe AD, Doyle RC, McDonald MM, Randi F, Lonergan P, Butler ST (2025). Mortality of calves derived from in vitro produced embryos..

[B046] Pfeffer PL, Pearton DJ (2012). Trophoblast development. Reproduction.

[B047] Pohler KG, Pereira MHC, Lopes FR, Lawrence JC, Keisler DH, Smith MF, Vasconcelos JLM, Green JA (2016). Circulating concentrations of bovine pregnancy-associated glycoproteins and late embryonic mortality in lactating dairy herds. J Dairy Sci.

[B048] Rabaglino MB, Bojsen-Møller Secher J, Sirard M-A, Hyttel P, Kadarmideen HN (2021). Epigenomic and transcriptomic analyses reveal early activation of the HPG axis in in vitro-produced male dairy calves. FASEB J.

[B049] Rabaglino MB, Secher JB, Hyttel P, Kadarmideen HN (2022). In vitro- and in vivo-produced male dairy calves show molecular differences in the hepatic and muscular energy regulation. Biol Reprod.

[B050] Ritter C, Beaver A, von Keyserlingk MAG (2019). The complex relationship between welfare and reproduction in cattle. Reprod Domest Anim.

[B051] Rizos D, Scully S, Kelly AK, Ealy AD, Moros R, Duffy P, Al Naib A, Forde N, Lonergan P (2012). Effects of human chorionic gonadotrophin administration on day 5 after oestrus on corpus luteum characteristics, circulating progesterone and conceptus elongation in cattle. Reprod Fertil Dev.

[B052] Rizos D, Ward F, Duffy P, Boland MP, Lonergan P (2002). Consequences of bovine oocyte maturation, fertilization or early embryo development in vitro versus in vivo: implications for blastocyst yield and blastocyst quality. Mol Reprod Dev.

[B053] Sánchez JM, Mathew DJ, Behura SK, Passaro C, Charpigny G, Butler ST, Spencer TE, Lonergan P (2019). Bovine endometrium responds differentially to age-matched short and long conceptusesdagger. Biol Reprod.

[B054] Santos A, Minela T, Branen J, Pursley JR (2023). Time to increase in pregnancy-specific protein B following artificial insemination is a direct determinant of subsequent pregnancy loss in lactating dairy cows. J Dairy Sci.

[B055] Sartori R, Bastos MR, Wiltbank MC (2010). Factors affecting fertilisation and early embryo quality in single- and superovulated dairy cattle. Reprod Fertil Dev.

[B056] Sasser RG, Ruder CA, Ivani KA, Butler JE, Hamilton WC (1986). Detection of pregnancy by radioimmunoassay of a novel pregnancy-specific protein in serum of cows and a profile of serum concentrations during gestation. Biol Reprod.

[B057] Scatolin GN, Ming H, Wang Y, Iyyappan R, Gutierrez-Castillo E, Zhu L, Sagheer M, Song C, Bondioli K, Jiang Z (2024). Single-cell transcriptional landscapes of bovine peri-implantation development. iScience.

[B058] Stangaferro ML, Toledo MZ, Gennari RS, Perez MM, Gamarra CA, Sitko EM, Monteiro PLJ, Masello M, Prata AB, Granados GE, Van Amburgh ME, Luchini D, Shaver RD, Wiltbank MC, Giordano JO (2021). Effects of feeding rumen-protected methionine pre- and postpartum on reproductive outcomes of multiparous Holstein cows. J Dairy Sci.

[B059] Thompson L, Crowe AD, Rabaglino MB, Butler ST, Lonergan P (2025). Sex-related changes in liver and muscle transcriptome of calves derived from artificial insemination or the transfer of an in vitro-produced embryo. Biol Reprod.

[B060] United Nations (2016). Sustainable development goals..

[B061] van Leeuwen J, Berg DK, Pfeffer PL (2015). Morphological and gene expression changes in cattle embryos from hatched blastocyst to early gastrulation stages after transfer of in vitro produced embryos. PLoS One.

[B062] Viana JHM (2023). 2022 Statistics of embryo production and transfer in domestic farm animals: the main trends for the world embryo industry still stand. Embryo Technology Newsletter.

[B063] Wathes DC, Wooding FB (1980). An electron microscopic study of implantation in the cow. Am J Anat.

[B064] Webb LE, Verwer C, Bokkers EAM (2023). The future of surplus dairy calves: an animal welfare perspective. Front. Anim. Sci..

[B065] Wiltbank MC, Baez GM, Garcia-Guerra A, Toledo MZ, Monteiro PL, Melo LF, Ochoa JC, Santos JE, Sartori R (2016). Pivotal periods for pregnancy loss during the first trimester of gestation in lactating dairy cows. Theriogenology.

[B066] Wooding FBP (2022). The ruminant placental trophoblast binucleate cell: an evolutionary breakthrough. Biol Reprod.

